# Carbapenemase-producing *Enterobacteriaceae* and *Aeromonas* spp. present in wastewater treatment plant effluent and nearby surface waters in the US

**DOI:** 10.1371/journal.pone.0218650

**Published:** 2019-06-26

**Authors:** Dimitria A. Mathys, Dixie F. Mollenkopf, Sydnee M. Feicht, Rachael J. Adams, Amy L. Albers, David M. Stuever, Susan V. Grooters, Gregory A. Ballash, Joshua B. Daniels, Thomas E. Wittum

**Affiliations:** 1 Department of Veterinary Preventive Medicine, College of Veterinary Medicine, The Ohio State University, Columbus, Ohio, United States of America; 2 Department of Veterinary Clinical Sciences, College of Veterinary Medicine, The Ohio State University, Columbus, Ohio, United States of America; Purdue University, UNITED STATES

## Abstract

Carbapenemase-producing bacteria (CPB) are rare, multidrug resistant organisms most commonly associated with hospitalized patients. Metropolitan wastewater treatment plants (WWTP) treat wastewater from large geographic areas which include hospitals and may serve as epidemiologic reservoirs for the maintenance or expansion of CPB that originate from hospitals and are ultimately discharged in treated effluent. However, little is known about the potential impact of these WWTP CPB on the local surface water and their risk to the public health. In addition, CPB that are present in surface water may ultimately disseminate to intensively-managed animal agriculture facilities where there is potential for amplification by extended-spectrum cephalosporins. To better understand the role of WWTPs in the dissemination of CPB in surface waters, we obtained samples of treated effluent, and both upstream and downstream nearby surface water from 50 WWTPs throughout the US. A total of 30 CPB with clinically-relevant genotypes were recovered from 15 WWTPs (30%) of which 13 (50%) serviced large metropolitan areas and 2 (8.3%) represented small rural populations (P < 0.05). Recovery of CPB was lowest among WWTPs that utilized ultraviolet radiation for primary disinfection (12%), and higher (P = 0.11) for WWTPs that used chlorination (42%) or that did not utilize disinfection (50%). We did not detect a difference in CPB recovery by sampling site, although fewer CPB were detected in upstream (8%) compared to effluent (20%) and downstream (18%) samples. Our results indicate that WWTP effluent and nearby surface waters in the US are routinely contaminated with CPB with clinically important genotypes including those producing *Klebsiella pneumoniae* carbapenemase (KPC) and New Delhi metallo-beta-lactamase (NDM). This is a concern for both public health and animal agriculture because introduction of CPB into intensively managed livestock populations could lead to their amplification and foodborne dissemination.

## Introduction

The therapeutic use of carbapenem antimicrobials has been followed by the emergence and dissemination of clinically-relevant carbapenemase-producing *Enterobacteriaceae* (CPE). In 2017, the World Health Organization (WHO) ranked *Enterobacteriaceae* resistant to carbapenems and extended-spectrum cephalosporins in the top tier of their new “priority pathogens” list of resistant bacteria for which research on new therapies are needed [1]. The Centers for Disease Control (CDC) has reported approximately 9,000 CPE infections annually in the US with an estimated mortality rate in some healthcare settings approaching 50% when they infect high-risk patient populations [2]. The *Klebsiella pneumoniae* carbapenemase gene, *bla*_KPC_, first emerged in human clinical isolates from the eastern US in 1996 and has since complicated therapeutic treatment in healthcare facilities throughout the US [3] and globally [4]. *bla*_KPC_ and other mobile carbapenemase genes, including the metallo β-lactamases, have migrated beyond the healthcare environment and are now recovered from both hospital- and community-acquired human infections in multiple bacterial species [5]. Problematically, these carbapenemase-producing bacteria may be disseminated in waste flows exiting healthcare facilities and have been reported in wastewater effluent and surface water [6].

Moving beyond human healthcare, some CPE including KPC-producing *Enterobacteriaceae* have been found in environmental matrices with potentially serious implications for the public health [7]. Effluent water samples collected in August and December 2008 at a hospital WWTP in metropolitan Rio de Janiero, Brazil carried *Klebsiella pneumonia* with *bla*_KPC-2_ [8]. Additional isolates producing KPC-2 were later recovered in 2013 from multiple *Enterobacteriaceae* and other bacteria including *Aeromonas* sp., *Citrobacter* sp., *Enterobacter* sp., *K*. *pneumoniae*, and *Kluyvera* sp. These isolates were collected from multiple recreational surface waters in Rio de Janiero [9,10]. In Europe, *Escherichia coli* ST410 harboring *bla*_KPC-2_ on an IncF plasmid were recovered in 2010 from water samples collected from a river which transects the city of Santo Tirso in Northern Portugal. Rarely reported even in endemic regions, this was the first report of KPC-producing *E*. *coli* in that country [11]. *E*. *coli* producing KPC-2 have also been recovered from a river ecosystem in Spain. Real-time PCR (qPCR) quantification of KPC-2 gene copies in hospital effluent from two facilities in the Catalonia region of northeastern Spain was 4.4x10^7^ and 5.4x10^4^ per milliliter of sample [12]. These findings highlight the concerning potential for waste-mediated dissemination of CPE originating from hospital settings moving into open public waterways, and demonstrate the potential for CPE to disseminate across large geographic regions. However, the extent of this environmental expansion of CPE remains unknown.

Our objective is to better understand the role of WWTPs in the dissemination of clinically-important antibiotic resistant bacteria into the environment in surface water. We have hypothesized that CPE commonly survive and grow in hospital waste as it is transported to municipal wastewater treatment plants, where they are reduced by treatment but survive and are discharged in effluent into surface waters. We expect CPE to be recovered more frequently from wastewater effluent and nearby surface water from treatment plants servicing population dense cities, such as those with landmarks including major healthcare facilities, compared to effluent from plants servicing rural/agricultural areas with lower population density. We expect that variability in wastewater treatment practices applied by WWTPs will impact the recovery of CPE from effluent and nearby surface waters. Also, we expect *bla*_KPC_-bearing isolates to be recovered more frequently from eastern US states, where this genotype is now endemic in healthcare environments, compared to other regions of the US [13].

## Materials and methods

To address our hypotheses, we identified two WWTPs in each of the 48 continental US states, one that served a large metropolitan area, and one that served a small, rural town. Only WWTPs that discharged into open waterways were selected where downstream sample collection was possible, and coastal WWTPs that discharged into ocean waters were excluded from our study. Metropolitan WWTPs were identified using a list of most populated US cities segregated by state (https://en.wikipedia.org/wiki/List_of_U.S._states%27_largest_cities_by_population) and local public utilities websites. Rural towns with WWTPs were identified using the search function of Google Maps. Preference was given to small towns of <5,000 people with a maximum population allowance of 10,000 residents.

WWTPs that agreed to participate were asked to provide samples of treated effluent water as well as surface water samples both upstream and downstream of effluent discharge. We did not request untreated influent samples from the participating WWTPs because our hypothesis was specific to resistant bacteria discharged into the environment in surface water. The WWTPs were each mailed a sampling kit that included sampling instructions, three pre-labeled 1 L sterile bottles for sampling, parafilm to seal the bottles, absorbent packing material, and sealable 4 L bags for each sample to prevent leakage during shipping. A brief questionnaire accompanied the kit to capture statistics regarding cubic meters processed per second (cms), area serviced by the plant (sq km) and its geographic location, distance from discharge to upstream and downstream collection (km), and at what water depth (m) those samples were collected. The WWTPs were also asked to provide their method of primary disinfection and concentration of residual ammonia in their effluent. Samples and information were provided voluntarily by authorized employees of each WWTP, and the investigators provided assurance that the names and locations of the participating WWTPs would remain confidential. However, the authors may be able to obtain permission to reveal the locations of individual participating WWTPs upon request.

For the recovery of CPE and other carbapenemase-producing bacteria (CPB), each 1 L water sample was vacuum filtered through a series of sterile filters (20 μm nylon net, 10 μm nylon net, and 0.8 μm cellulose, MilliporeSigma, Burlington MA) intended to efficiently remove liquid while capturing progressively smaller particles, culminating in a 0.45 μm pore size cellulose filter (Fisher Scientific, Hampton NH) to capture bacteria. All filters were combined and incubated overnight at 37°C in 100 ml of MacConkey broth modified with 0.5 μg/ml of meropenem and 70 μg/ml of zinc sulfate, with the broth then inoculated onto similarly enriched MacConkey agar and incubated overnight at 37°C to identify isolates expressing phenotypic carbapenem-resistance [14,15]. Isolates representing unique bacterial morphologies were selected from each agar plate with priority given to lactose fermenting colonies. Carbapenemase production was assessed using Carba NP [16] with bacterial speciation accomplished using MALDI-TOF mass spectrometry (Biotyper, Bruker Daltonics, Billerica, MA). Intrinsically carbapenem resistant bacterial species including *Pseudomonas otitidis* and *Stenotrophomonas maltophilia* that are commonly present in soil and water and expected to harbor chromosomally encoded carbapenemase genes were excluded from further analysis. The remaining carbapenemase-producing isolates were characterized using whole genome sequencing (WGS; MiSeq; Illumina, San Diego, CA). The data have been deposited with links to BioProject accession number PRJNA472583 in the NCBI BioProject database (https://www.ncbi.nlm.nih.gov/bioproject/). Isolates not identified at the species level by MALDI-TOF were identified using KmerFinder 2.0 [17,18]. Plasmid and β-lactamase gene content was evaluated using PlasmidFinder 1.3 [19], ResFinder 3.0 [20], CARD 2.0.2 [21], and ARG-ANNOT V4 [22]. Multilocus sequence types were determined using the MLST 2.0 database [23].

Ordinary logistic regression (OLR) was used to compare the proportion of WWTPs from which one or more samples produced CPB between metropolitan and rural plants, between plants using chlorination or ultraviolet radiation for primary disinfection, and to compare the proportion of samples that produced CPB from the three different sampling sites. In addition, OLR was used to investigate the association of ammonia concentration in effluent with the probability of CPB recovery.

## Results

**Fig 1:**
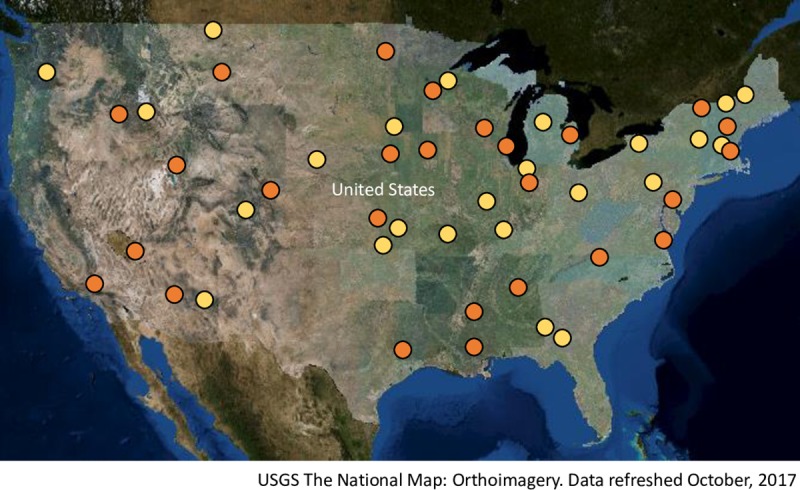
Locations of 50 US wastewater treatment plants participating in this study by providing effluent, upstream, and downstream water samples. Plants servicing large metropolitan areas (mean population > 800,000) are shown in orange and plants servicing rural small towns (population < 10,000) are shown in yellow.

We received effluent, upstream, and downstream surface water samples from 50 US WWTPs (response rate = 52%) between July and December of 2016 (Fig 1). Upstream samples were taken at a mean distance of 1.85 km from discharge with downstream samples collected a mean 1.59 km away from discharge. Samples were collected at a mean depth of 0.48 m below the water surface. Twenty-six sets of water samples were from WWTPs servicing large metropolitan cities with a mean population of over 800,000 residents. These plants treated an average daily wastewater flow of 3.5 cms with an average service area of 496 sq km. The remaining 24 sets of samples were from plants servicing small rural towns with a mean population of 5,203 residents. These plants treated a daily flow of 0.5 cms of wastewater on average and serviced an average area of 36 sq km. Of the 47 WWTPs that provided information about their disinfection practices, 26 (55%) reported that they used chlorination, 17 (37%) reported that they used ultraviolet radiation, and 4 (9%) reported that they did not utilize disinfection. Thirty-eight of the WWTPs provided their ammonia concentrations in effluent with a reported mean of 3.4 mg/L (sd = 5.7) and ranging from 0.1 to 22.6 mg/L. Among the 19 WWTPs using chlorination for primary disinfection and also reported ammonia concentration in effluent, 9 (47%) reported negligible residual ammonia (<2.0 mg/L) indicating the use of free chlorine. The remaining 10 WWTPs (53%) reported higher residual ammonia (≥ 2.0 mg/L) indicating the use of chloramine.

We recovered 243 bacterial isolates with reduced susceptibility to meropenem, of which 90 exhibited the ability to hydrolyze carbapenem antimicrobials. These resulting 90 carbapenemase-producing isolates represented water samples collected from 35 treatment facilities– 20 metropolitan and 15 rural plants. From these, 13 isolates were identified by MALDI-TOF as *P*. *otitidis* which are commonly present in soil and water and are expected to express intrinsic carbapenem resistance [24]. These isolates were excluded from further characterization because they are unlikely to produce human infections that require antibiotic therapy and they do not typically mobilize their carbapenemase genes [25]. Thirty-one *Aeromonas* spp. isolates were also expected to express a chromosomally encoded carbapenemase gene [26], but have been reported to acquire additional mobile carbapenemase genes [27]. Therefore, these *Aeromonas* spp. isolates were evaluated for the carriage of acquired carbapenemase genes by conventional single-plex PCR using primer pairs for the five carbapenemase genes of greatest clinical relevance in the US–*bla*_KPC_, *bla*_NDM_, *bla*_IMP_, *bla*_VIM_, and *bla*_OXA_ [28–31]. Of the 31 *Aeromonas* spp. isolates, 5 representing distinct WWTPs carried *bla*_KPC_, with no other carbapenemase genes detected by conventional PCR. The five *bla*_KPC_-bearing *Aeromonas* isolates were further characterized using WGS.

An additional 19 intrinsically carbapenem-resistant soil- and water-associated isolates which could not fully be identified by MALDI-TOF were identified by WGS using KmerFinder 2.0 [17,18] and excluded from further characterization because they are unlikely to produce human infections requiring antibiotic therapy, and they typically do not mobilize their carbapenemase genes. These isolates included 12 *P*. *resinovorans*, 5 *Chromobacterium violaceum*, 1 *P*. *alcaligenes*, and 1 *P*. *otitidis*. Two isolates, 1 *P*. *aeruginosa* strain PAO and 1 *P*. *putida* identified by KmerFinder, appeared to produce carbapenemase based on the Carba NP result, but no known carbapenemase genes could be identified using currently available databases and so were excluded from this analysis.

**Fig 2:**
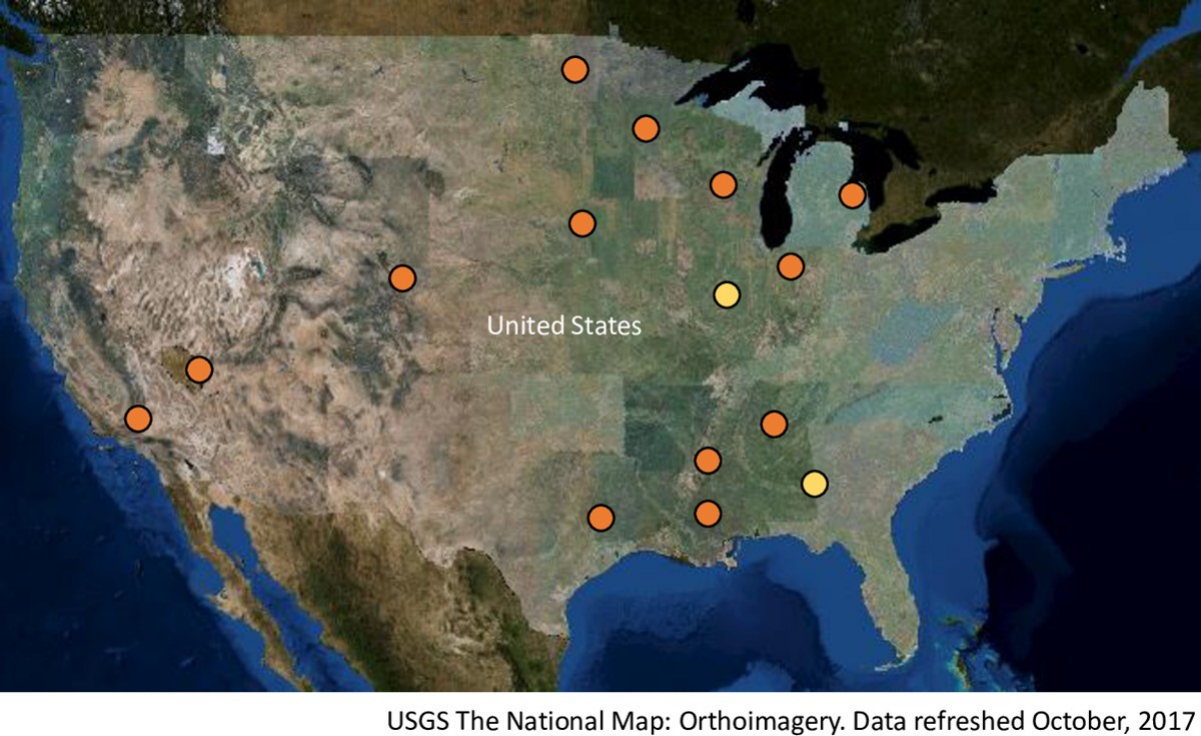
Locations of 15 US wastewater treatment plants from which clinically-relevant carbapenemase-producing bacteria were recovered from effluent, upstream, or downstream water samples. Plants servicing large metropolitan areas (mean population > 800,000) are shown in orange and plants servicing rural small towns (population < 10,000) are shown in yellow.

Carbapenemase genes and plasmid content were identified for the remaining 30 bacteria with clinically-relevant resistance genotypes (Table 1) representing effluent and surface water sampled from 15 WWTPs using WGS. Our recovery ranged from a single CPB isolate per plant to as many as seven diverse isolates from a single wastewater treatment facility. While most samples yielded a single CPB isolate, one upstream sample contributed three non-clonal CPB isolates. In total, these CPB isolates were filtered from 23 water samples which included 8% of upstream samples (n = 4), 18% of effluent samples (n = 9), and 20% of downstream samples (n = 10), and we could not detect a difference in CPE recovery by sampling site. With the exception of two WWTPs located on the West coast and a plant on the western edge of the Great Plains, the plants were located in the central US (Fig 2). Thirteen (50%) of the 26 treatment plants servicing large metropolitan areas produced one or more CPB isolates, while only two (8.3%) of the plants representing small rural towns (n = 24) produced a single CPB isolate each (P < 0.05; Table 1).

**Table 1:** Characteristics of 30 carbapenemase-producing bacteria recovered from wastewater treatment plant effluent, or from nearby upstream or downstream surface water in the US.

WWTP	State	Metro or Rural	Collection date	Sample type^a^	Genus and species identification	Bacterial sequence type^c^	Carbapenemase	Other β-lactamases	Plasmid content	GenBank Accession Number
4	GA	Rural	7/13/2016	Effluent	*Enterobacter cloacae* complex sp.	ST-928	KPC-2	CARB-2, FOX-5, OXA-1	IncA/C2, IncN, IncP6, RepA	SAMN09288744
6	IL	Rural	7/14/2016	Down	*Raoultella ornithinolytica*	NA	GES-5	ORN-1b	IncP6, IncR	SAMN09289736
16	NE	Metro	8/2/2016	Effluent	*Citrobacter freundii*	ST-413	KPC-3	SHV-12, TEM^d^	IncFIB, IncHI2A, Col440I	SAMN09289737
20	CA	Metro	8/15/2016	Down	*Escherichia coli*	ST-410	NDM-1	CTX-M-15, OXA-1	IncA/C2, IncFIB, IncY, Col(BS512)	SAMN09289738
21	ND	Metro	8/15/2016	Effluent	*Citrobacter freundii*	ST-11	KPC-3	CMY-66, SHV-12, TEM-1B	Col440I, IncX3	SAMN09289739
23	AL	Metro	8/16/2016	Down	*Enterobacter cloacae*	ST-1121	KPC-2	SHV^d^	FIB(pQil), IncL/M(pMU407), IncFII(Yp)	SAMN09289740
23	AL	Metro	8/16/2016	Down	*Aeromonas caviae*^b^	ST-560	KPC-2	OXA-9, TEM^d^	IncP6	SAMN09289741
28	NV	Metro	8/29/2016	Down	*Klebsiella pneumoniae*	ST-17	NDM-7	CTX-M-15, SHV-11, TEM-1B	IncFIB, IncX3	SAMN09289742
28	NV	Metro	8/29/2016	Up	*Klebsiella quasipneumoniae*	ST-138	KPC-2	SHV-12, OKP-B-2	IncA/C2, IncFIB(K)	SAMN09289743
30	LA	Metro	9/19/2016	Effluent	*Enterobacter cloacae* complex sp.	ST-595	KPC-2, GES-5	MIR-6	IncFIB(pECLA), IncP6, RepA_1_pKPC	SAMN09289744
33	MS	Metro	9/26/2016	Down	*Klebsiella pneumoniae*	ST-3539	KPC-2	OKP-B-7	IncFIB(K), IncFII(K), IncP6	SAMN09289745
33	MS	Metro	9/26/2016	Effluent	*Enterobacter cloacae*	ST-41	KPC-2	OXA-2, MIR-3	IncX5, IncFII(Yp)	SAMN09289746
33	MS	Metro	9/26/2016	Up	*Klebsiella oxytoca*	ST-88	KPC-2	CARB-2, OXY-1^d^, SHV^d^	IncA/C2, IncFIB(K), IncL/M	SAMN09289747
37	CO	Metro	10/11/2016	Down	*Aeromonas caviae*^b^	ST-561	KPC-2		IncP6	SAMN09289748
39	MN	Metro	10/17/2016	Effluent	*Aeromonas caviae*^b^	ST-562	KPC-2			SAMN09289749
45	TX	Metro	10/31/2016	Down	*Enterobacter cloacae*	ST-1122	KPC-2	TEM^d^	IncFIA(HI1), IncFIB, IncQ2	SAMN09289750
47	IN	Metro	11/14/2016	Down	*Raoultella planticola*	NA	VIM-1	PLA2^d^	IncA/C2, IncFIB(K), IncFII	SAMN09289751
47	IN	Metro	11/14/2016	Effluent	*Enterobacter asburiae*	ST-24	KPC-2	ACT-1, TEM-1	IncFIB(pECLA), IncFII(pECLA), IncHI2A, IncP6	SAMN09289753
47	IN	Metro	11/14/2016	Effluent	*Escherichia coli*	ST-607	KPC-3	AmpC1, AmpC2	IncA/C2, IncW	SAMN09289752
47	IN	Metro	11/14/2016	Up	*Aeromonas caviae*^b^	ST-563	KPC-2	OXA-105	IncP6	SAMN09289754
48	WI	Metro	11/14/2016	Down	*Raoultella ornithinolytica*	NA	KPC-2	OXA-1, FOX-5, CARB-2, ORN1b	IncA/C2, IncP6	SAMN09289755
48	WI	Metro	11/14/2016	Effluent	*Citrobacter freundii*	ST-8	KPC-2	CMY-79, FOX-5, CARB-2	IncA/C2, IncX5	SAMN09289757
48	WI	Metro	11/14/2016	Effluent	*Aeromonas caviae*^b^	ST-564	KPC-2	OXA-2	IncP6, IncQ2	SAMN09289756
49	MI	Metro	11/16/2016	Down	*Klebsiella oxytoca*	ST-127	KPC-2	TEM-1B, OXY-5	IncFIB, IncFII, Col440I	SAMN09289758
49	MI	Metro	11/16/2016	Down	*Klebsiella pneumonia*	ST-872	KPC-2	TEM-1B, SHV-11	IncFIB(Mar), IncFII, IncN	SAMN09289759
49	MI	Metro	11/16/2016	Effluent	*Enterobacter cloacae* complex sp.	ST-131	KPC-2	TEM-1A, OXA-9, MIR-9, CARB-2	IncA/C2, IncFIB(K), IncFII, IncR, repA_1_pKPC-2, Col440I	SAMN09289761
49	MI	Metro	11/16/2016	Effluent	*Escherichia coli*	ST-167	NDM-5	AmpC1, OXA-1	IncFII	SAMN09289760
49	MI	Metro	11/16/2016	Up	*Enterobacter cloacae* complex sp.	ST-1028	KPC-2	TEM-1B, SHV-12	IncFIB(pECLA), IncHI1A(CIT), repA_1_KPC-2	SAMN09289763
49	MI	Metro	11/16/2016	Up	*Enterobacter cloacae* complex sp.	ST-984	KPC-2	TEM-1B, MIR-15	IncFIB(pECLA), IncFII(pCRY), repA_1_KPC-2	SAMN09289762
49	MI	Metro	11/16/2016	Up	*Klebsiella pneumoniae*	ST-2793	KPC-2	TEM-1B, SHV^d^	IncFIB, IncHI1B_1_pNDM-Mar, repA_1_KPC-2	SAMN09289764

^a^ Effluent = wastewater treatment plant treated discharge; Up = Surface water upstream of WWTP discharge; Down = Surface water downstream of WWTP discharge

^b^ *Aeromonas caviae* identified by MALDI-TOF, but species could not be confirmed using KmerFinder online database at the Center for Genomic Epidemiology website.

^c^ NA = Sequence typing scheme not currently available for *Raoultella* spp.

^d^ The specific allele could not be identified using currently available antibiotic resistance gene databases.

One or more CPB isolates were recovered from 2 of the 4 (50%) WWTPs that reported they did not use disinfection, from 11 of the 26 (42%) WWTPs using chlorination for disinfection, but from only 2 of 17 (12%) WWTPs using ultraviolet radiation for disinfection. However, we could not detect this difference (P = 0.11) when adjusted for the potential confounding effects of metropolitan vs. rural service area. The recovery of CPB was not associated with the reported concentration of ammonia in effluent. Mean reported ammonia concentration was 3.0 mg/L (sd = 5.6; n = 25) for WWTPs from which we did not recover CPB, and 4.2 mg/L (sd = 5.9; n = 13) for WWTPs from which one or more CPB were recovered. Among WWTPs using chlorination for disinfection that also reported ammonia in effluent (n = 19), CPB recovery was similar between those that reported residual ammonia concentrations indicating the use of free chlorine (44%; n = 9) vs chloramine (50%; n = 10).

Twenty-two (73.3%) of the 30 CPB, comprised of 5 *A*. *caviae*, 1 *Citrobacter freundii*, 9 *Enterobacter* spp, 6 *Klebsiella* spp and a *Raoultella ornithinolytica*, harbored *bla*_KPC-2_, with 2 additional *C*. *freundii* and an *E*. *coli* carrying *bla*_KPC-3_. Single *E*. *coli* isolates carried *bla*_NDM-1_ and *bla*_NDM-5_, respectively, while *bla*_NDM-7_ was detected in one *K*. *pneumoniae*. A second *R*. *ornithinolytica* isolate harbored *bla*_GES-5_ and one *R*. *planticola* was identified with *bla*_VIM-1_. With the exception of a single *E*. *coli*, CPB isolates carried additional β-lactamase genes, the most prevalent being *bla*_TEM_ and *bla*_SHV_ (Table 1). One *E*. *cloacae* ST 595 isolate carried two mobile Ambler class A carbapenemase genes, the prevalent *bla*_KPC-2_ and a *bla*_GES-5_ along with an AmpC *bla*_MIR-6_.

Examination of plasmid content found most CPB possessed multiple plasmids, frequently one IncF plasmid along with other plasmids of diverse incompatibility (Table 1). Plasmid content, detected at a 90% similarity threshold using the Center for Genomic Epidemiology’s PlasmidFinder online database [19], ranged from none to as many as 6 plasmids identified per isolate.

## Discussion

US WWTP effluent, upstream, and downstream surface water samples yielded a diverse mixture of carbapenemase-producing bacterial species, carbapenem-resistance genotypes, and plasmid replicon types. As expected, most of these isolates (66.7%) represented bacteria with expected, intrinsic carbapenem-resistance, frequently found in soil and water environments. These CPB can produce serious disease in individual patients but are not epidemiologically relevant because they rarely produce human infections [32], are not normal residents of patient flora [25,32], and because they do not typically mobilize carbapenemase-encoding genetic elements [25]. However, thirty bacterial isolates (33.3%) represented highly clinically relevant carbapenem-resistant genotypes reported with increasing frequency to cause nosocomial infections that are often associated with negative patient outcomes [33]. These clinically-relevant CPB isolates were comprised of 8 bacterial species including *E*. *cloacae* and *K*. *pneumoniae*, both of which have disseminated as epidemics in healthcare environments [34–36]. *bla*_KPC-2_ was the carbapenemase gene we found most frequently (73.3%) among these bacteria that were recovered using selective enrichment, reflecting its status as the most prevalent carbapenemase gene reported in US clinical infections [37].

CPB of clinical concern were more frequently recovered from urban WWTPs servicing major metropolitan areas, with 13 of the 15 WWTPs (86.7%) which yielded CPB with mobile carbapenemase genes located in large cities compared to only 2 CPB producing WWTPs (13.3%) in small rural towns. Large metropolitan cities with mean populations of over 800,000 residents could be expected to have at least one major hospital and multiple long-term care and skilled nursing facilities. These facilities that combine high-risk patient populations, heightened colonization pressure, and potential exposure to carbapenems may serve as reservoirs for CPB which exit the facilities through wastewater flows, making their way to local WWTPs [38–40].

One or more CPB were recovered from 42% of WWTPs reporting that they used chlorination for disinfection, compared to only 12% of WWTPs reporting disinfection using ultraviolet radiation. While we could not detect this difference, the lower observed CPB recovery suggests that additional investigation of the relative ability of ultraviolet radiation disinfection to reduce antibiotic resistant bacteria in WWTP effluent compared to chlorination may be warranted. We did not observe an association between ammonia concentration in effluent and the recovery of CPB, although our low response rate (76%; n = 38) likely limited our statistical power to detect a difference. More importantly, we observed only a slightly lower recovery of CPB from WWTPs with residual ammonia indicating the use of free chlorine compared to chloramine, although the low number of observations (n = 19) again likely limited our statistical power to detect a difference. However, our observed differences were small and provide little justification for additional investigation of this potential association.

We did not detect a difference in the proportion of samples that yielded clinically-relevant CPB between WWTP effluent and upstream or downstream surface water samples. This result suggests that CPB carried in the effluent may disseminate both upstream and downstream around the discharge, and not just move downstream with the water flow. The recovery of CPB upstream of the effluent discharge may also represent WWTPs or other sources of contamination upstream of our sampling sites which might include other WWTPs, combined stormwater/sewer overflows, or sanitary sewer overflows. It is also important to note that we did not quantify CPB in the samples, and it is possible that CPB concentrations in the effluent, upstream, and downstream samples differed. The two CPB-positive rural WWTPs yielded only a single isolate each while metropolitan plants were responsible for as many as seven novel carbapenemase-producing isolates per plant. Although these isolates represent diverse bacterial species, carbapenemase genes, and plasmid combinations, some may be a result of our selective culture, but the mobilizable or transposable resistance elements still needed to be present in that sample.

The predominant CPB globally, *bla*_KPC-2_, was first reported in a patient in North Carolina in 1996 and quickly disseminated in healthcare settings in the eastern US [37]. Surprisingly, we did not recover any CPB isolates from WWTP on the US east coast. With the exception of two WWTPs located in the southwestern US and a single facility in the Plains region, the WWTPs with clinically-relevant CPB isolates were geographically clustered in the central US in a broad column reaching as far west as the Great Plains. For inclusion in our study we specified that each WWTP needed to collect both an upstream and downstream surface water sample in addition to the effluent. Many coastal locations discharge into ocean waters making an accurate downstream sample often unattainable, and so were excluded from our study. This sampling criteria may have limited our ability to identify CPB in population dense cities along the east and west coast. The lack of CPB isolates from the Plains and Rocky Mountain regions of the US may be reflective of both low population density and fewer WWTPs sampled. In addition, in arid and semi-arid portions of the country, drought and population growth of the last century has led to water shortages and an increase in wastewater reclamation and reuse in those regions, thus limiting the number of potential WWTP discharging into open waterways [41]. Also, differences in state and local regulations regarding treatment and the quality of WWTP effluent likely influenced our observed results. In particular, stricter requirements for wastewater nutrient removal in states on the east and west coast of the US attempting to protect coastal waterways may be an explanation for the low number of CPB we recovered from these regions. However, we did not collect data on differences in regional or state regulatory requirements for WWTP treatment practices and effluent for this study.

The dissemination of clinically-relevant antibiotic-resistant bacteria such as CPB into the environment poses a direct threat to the public health as a potential reservoir for community-acquired infections. In addition, uncontrolled environmental dissemination will inevitably lead to the introduction of CPB into intensively managed agricultural animal populations [15,42] where they may threaten animal health and agricultural productivity. In addition, the frequent application of extended-spectrum cephalosporin antibiotics in animal agriculture [43] may result in the expansion of CPB in animals preparing to enter the food supply as fresh meat products [14]. Because antibiotic-resistant enteric bacteria from food animals commonly contaminate fresh retail meat products [44,45], this potential for expansion of CPB in livestock suggests the possibility of foodborne transmission.

These data indicate that clinically-relevant CPB commonly associated with healthcare currently contaminate both WWTP effluent and nearby surface waters in the US. This signals a clear need for mediation efforts to remove or reduce the public health threat posed by these highly resistant bacterial strains in public waterways. Care must be taken when implementing such efforts to assure negative impacts on the environment are minimized. Additional research is likely warranted to identify the most effective intervention points and methodologies to reduce the environmental dissemination and expansion of CPB.

## Supporting information

S1 Table Descriptive data collected for 50 US wastewater treatment plants that provided samples of treated effluent, and upstream and downstream surface water samples to be tested for recovery of carbapenemase-producing bacteria. (DOCX)
