# Supplementary material for: Carbapenemase-producing Enterobacteriaceae and Aeromonas spp. present in wastewater treatment plant effluent and nearby surface waters in the US
Source: PLoS One. 2019 Jun 26;14(6):e0218650. doi: 10.1371/journal.pone.0218650 (PMC6594618; doi:10.1371/journal.pone.0218650)
Supplement: S1 Table — (DOCX) [file pone.0218650.s001.docx]

**Table S1**. Descriptive data collected for 50 US wastewater treatment plants that provided samples of treated effluent, and upstream and downstream surface water samples to be tested for recovery of carbapenemase-producing bacteria.

| **WWTP** | **CPB status^a^** | **Disinfection type^b^** | **Ammonia (mg/L)^c^** | **State** | **Metro or Rural** | **Collection date** | **Volume (m^3^/s)^d^** | **Area (km^2^)^e^** | **Distance UP (km)^f^** | **Distance DOWN (km)^g^** | **Sample depth (m)** |
| --- | --- | --- | --- | --- | --- | --- | --- | --- | --- | --- | --- |
| 1 | Negative | Chorination |  | NH | Metro | 7/12/2016 | 0.88 | 56.29 | 4.60 | 0.35 |  |
| 2 | Negative | Chorination | 1.6 | RI | Metro | 7/12/2016 | 2.03 | 49.08 | 0.71 | 0.61 | 0.49 |
| 3 | Negative | UV | 0.1 | ID | Rural | 7/13/2016 | 0.07 | 11.27 | 0.10 | 0.13 | 0.30 |
| 4 | Positive | UV | 2.5 | GA | Rural | 7/13/2016 | 0.15 | 40.23 | 0.80 | 0.80 | 0.30 |
| 5 | Negative | Chorination | 8 | NH | Rural | 7/13/2016 | 0.01 | 1.26 | 0.18 | 0.05 | 0 |
| 6 | Positive | None | 0.2 | IL | Rural |  | 0.08 | 8.05 | 0.05 | 0.05 | 0.61 |
| 7 | Negative | UV | 0.5 | MT | Rural | 7/18/2016 | 0.01 | 1.61 | 0.18 | 0.18 | 0 |
| 8 | Negative | UV | 0.3 | MO | Rural | 7/18/2016 | 0.02 | 48.28 | 6.44 | 0.20 | 0.15 |
| 9 | Negative | UV | 4.5 | KS | Rural | 7/25/2016 | 0.08 | 14.48 | 1.77 | 0.80 | 0.08 |
| 10 | Negative | None | 5.5 | NY | Rural | 7/25/2016 | 0.06 | 3.38 | 0.00 | 0.01 | 0.30 |
| 11 | Negative | UV | 0.1 | CO | Rural | 7/26/2016 | 0.11 |  | 0.11 | 0.10 | 0.91 |
| 12 | Negative | UV | 1 | KS | Metro | 7/26/2016 | 1.74 | 90.33 | 2.35 | 0.92 | 0.23 |
| 13 | Negative | UV | 0.41 | IN | Rural | 7/27/2016 | 0.02 | 4.83 | 0.05 | 0.05 | 0 |
| 14 | Negative | Chorination | 0.4 | IL | Metro | 7/27/2016 | 13.15 | 482.79 | 1.61 | 2.09 | 0 |
| 15 | Negative | UV | 0.1 | NC | Metro | 8/1/2016 | 3.37 |  | 4.83 | 0 |  |
| 16 | Positive | Chorination | 14 | NE | Metro | 8/2/2016 | 3.37 | 482.79 | 0.03 | 0.21 | 1.22 |
| 17 | Negative | Chorination |  | MT | Metro | 8/8/2016 | 0.84 | 70.00 | 3.54 | 0.80 | 0 |
| 18 | Negative | UV | 0.2 | OR | Rural | 8/10/2016 | 0.06 | 32.19 | 0.02 | 0.02 | 6.10 |
| 19 | Negative | Chorination | 0.2 | IA | Metro | 8/15/2016 | 2.63 | 492.45 | 5.47 | 6.10 | 0.30 |
| 20 | Positive | Chorination |  | CA | Metro | 8/15/2016 | 5.26 |  |  |  |  |
| 21 | Positive | Chorination | 1 | ND | Metro | 8/15/2016 | 0.66 | 78.57 | 3.22 | 0.40 | 0.91 |
| 22 | Negative | UV |  | UT | Metro | 8/16/2016 | 2.63 | 185.07 | 0.05 | 0.05 | 0.23 |
| 23 | Positive | Chorination | 10 | AL | Metro | 8/16/2016 | 3.16 | 95.27 | 4.83 | 3.22 |  |
| 24 | Negative | None | 0.5 | IA | Rural | 8/17/2016 | 0.10 | 13.73 | 0.51 | 0.95 | 0 |
| 25 | Negative | UV | 1 | CT | Rural | 8/22/2016 | 0.05 | 103.00 | 0.10 | 0.55 | 0.12 |
| 26 | Negative | Chorination |  | AZ | Metro | 8/22/2016 | 6.84 | 1532.05 |  | 4.02 | 0.15 |
| 27 | Negative | Chorination | 0.1 | OH | Rural | 8/29/2016 | 0.12 | 13.66 | 0.31 | 2.41 |  |
| 28 | Positive | Chorination | 0.1 | NV | Metro | 8/29/2016 | 2.10 | 218.86 | 0.40 | 0.40 | 0 |
| 29 | Negative | Chorination |  | MA | Rural | 8/31/2016 | 0.05 | 56.33 | 0.14 | 1.61 | 0.76 |
| 30 | Positive | Chorination |  | LA | Metro | 9/19/2016 | 5.15 |  | 0.06 | 0.06 | 0.30 |
| 31 | Negative | Chorination | 6 | VT | Metro | 9/21/2016 | 0.03 | 1.13 | 0.05 | 0.03 | 0 |
| 32 | Negative | UV | 0.04 | ID | Metro | 9/26/2016 | 0.53 |  | 0.34 | 3.78 |  |
| 33 | Positive | Chorination | 0.1 | MS | Metro | 9/26/2016 | 2.42 | 233.35 | 1.61 | 1.61 | 0.91 |
| 34 | Negative | UV | 2 | NE | Rural | 9/27/2016 | 0.04 | 43.45 | 0.80 | 0 | 0 |
| 35 | Negative | Chorination | 2.2 | MI | Rural | 10/3/2016 | 0.06 | 6.44 | 0.80 | 1.61 | 0.30 |
| 36 | Negative | Chorination | 22.6 | ME | Rural | 10/5/2016 | 0.02 | 25.75 | 1.61 | 9.66 | 0.08 |
| 37 | Positive | Chorination | 1.7 | CO | Metro | 10/11/2016 | 1.18 | 173.80 | 1.13 | 0.64 | 0.15 |
| 38 | Negative |  |  | FL | Rural | 10/11/2016 | 0.03 | 8.05 | 0.45 | 0.32 | 0.20 |
| 39 | Positive | Chorination | 3.5 | MN | Metro | 10/17/2016 | 13.20 |  | 4.99 | 6.92 |  |
| 40 | Negative | Chorination | 17.5 | WI | Rural | 10/17/2016 | 0.08 | 15.61 | 0.02 | 0.16 | 0.30 |
| 41 | Negative | UV |  | DE | Metro | 10/18/2016 | 0.68 | 885.12 | 20.92 | 4.83 | 0.08 |
| 42 | Negative | Chorination |  | VA | Metro | 10/19/2016 | 0.74 | 72.42 | 3.22 | 8.05 |  |
| 43 | Negative | Chorination | 0.1 | PA | Rural | 10/25/2016 | 0.06 |  | 0.06 | 0.06 |  |
| 44 | Negative | UV | 0.25 | KY | Rural | 10/31/2016 | 0.01 | 4.62 | 0.80 | 0.80 |  |
| 45 | Positive | Chorination | 0.1 | TX | Metro | 10/31/2016 | 0.39 | 16.09 | 0.03 | 0.03 | 0.76 |
| 46 | Negative |  |  | OK | Rural | 11/7/2016 | 0.01 | 2.41 | 0.80 | 0.40 | 0 |
| 47 | Positive | UV | 1 | IN | Metro | 11/14/2016 | 5.00 | 598.66 | 2.41 | 2.41 | 2.44 |
| 48 | Positive | None | 2.6 | WI | Metro | 11/14/2016 | 2.10 | 289.67 | 0.74 | 2.61 | 0.15 |
| 49 | Positive | Chorination | 18 | MI | Metro | 11/16/2016 | 34.19 | 643.72 | 4.83 | 6.44 | 0.30 |
| 50 | Negative |  |  | AZ | Rural | 12/27/2016 | 0.05 | 32.19 | 0.80 | 0.80 |  |
|  | | |  |  |  |  |  |  |  |  |  |

^a^ Results of selective culture for the recovery of carbapenemase-producing bacteria from one or more samples

^b^ Reported method of primary disinfection; UV = ultraviolet radiation; empty cells indicate that data were not reported

^c^ Reported residual ammonia concentration in treated effluent; empty cells indicate that data were not reported

^d^ Volume of wastewater processed per day

^e^ Plant geographical service area; empty cells indicate that data were not reported

^f^ Distance of upstream surface water sample location from plant outfall; empty cells indicate that data were not reported

^g^ Distance of downstream surface water sample from plant outfall; empty cells indicate that data were not reported
